# Olmesartan-Induced Enteropathy: A Report of an Unusual Cause of Chronic Diarrhea

**DOI:** 10.7759/cureus.17004

**Published:** 2021-08-08

**Authors:** Christos Sotiropoulos, Eftichia Sakka, Georgia Diamantopoulou, Georgios J Theocharis, Konstantinos C Thomopoulos

**Affiliations:** 1 Gastroenterology Department, General University Hospital of Patras, Patras, GRC; 2 Internal Medicine Department, General University Hospital of Patras, Patras, GRC

**Keywords:** olmesartan, angiotensin receptor blockers, antihypertensive drugs, chronic diarrhea, sprue-like enteropathy

## Abstract

Olmesartan, an angiotensin II receptor blocker indicated in the treatment of hypertension, has been associ­ated with a seronegative sprue-like enteropathy that should be considered in the differential diagnosis of patients with unexplained chronic diarrhoea. It typically presents with severe chronic diarrhoea, considerable weight loss, and villous atrophy on biopsy and may be difficult to recognize because of its clinical and histological similarities to other clinical entities. Practically, discontinuation of the drug leads to dramatic recovery of the symptoms. We report a 76-year-old Caucasian female who was admitted to our hospital with complaints of chronic diarrhea and significant weight loss. Medical history was notable for hypertension being treated with olmesartan. Initially, investigation for all potential infectious causes and celiac disease was negative. Both upper and lower endoscopy was performed with duodenal biopsies revealing total villous atrophy and colonic biopsies showing lymphocytic colitis. In the presence of negative serology for celiac disease and after a thorough review of the patient’s medications, olmesartan in­duced-enteropathy was the most possible diagnosis. Olmesartan was discontinued and the symptoms rapidly resolved. A follow-up done a few months later showed no recurrence of the symptoms. In olmesartan-associated enteropathy, discontinuation of olmesartan results in immediate clinical recovery. Although rare, it is considered an emerging and underdiagnosed enteropathy. This case report illustrates the need for a thorough medication history evaluation and regular review during workup. We aim to increase the awareness of olmesartan-induced enteropathy among clinicians and gastroenterologists. We hope it will add to the current literature and help to understand this rare phenomenon in order to avoid unnecessary testing.

## Introduction

Olmesartan is an oral angiotensin receptor blocker (ARB) commonly prescribed in the management of hypertension, approved for use by the Therapeutic Goods Administration (TGA) since 2005 [1-3]. It blocks the angiotensin II receptor and is usually well-tolerated except for minor side effects, such as dizziness, influenza-like symptoms, and headache [1, 4].

Olmesartan-induced enteropathy (OIE) typically presents with a constellation of signs and symptoms, including chronic severe non-bloody diarrhea and significant weight loss that is unresponsive to a gluten-free diet [1-2, 5-6]. The time between olmesartan exposure and the onset of symptoms usually ranges from few months to five years [1]. Although the exact mechanism remains unknown, the clinical and histological findings are suggestive of a cell-mediated immune response [1].

Laboratory investigation commonly reveals findings consistent with a severe malabsorption process like anemia, electrolyte imbalance (hypokalemia and hypocalcemia), and hypoalbuminemia [1]. Typically, celiac serology (including anti-transglutaminase, anti-gliadin, and anti-endomysial antibodies) is always negative, antinuclear antibodies may be positive, and the majority of patients may have either HLA-DQ2 or DQ8 haplotypes [1, 4-5]. Histopathological findings include total or partial duodenal villous atrophy, mucosal granulocytic infiltration, and a thickened subepithelial collagen layer [1, 6].

We report the case of a 76-year-old patient with a non-coeliac, sprue-like enteropathy that was only resolved by ceasing olmesartan therapy. Our case report aims to increase the awareness among physi­cians of olmesartan-induced enteropathy.

## Case presentation

A 76-year-old woman, with a medical history of hypertension (treated with olmesartan for two years), anxiety disorder, and glaucoma, was referred to the Emergency Department due to a 12-month history of severe, non-bloody diarrhea (five to six watery stools per day without abdominal pain or fever) and unintentional weight loss of about 20 kg over the past six months. She reported a previous evaluation one year earlier, including an incomplete colonoscopy (up to the splenic flexure where the examination ended due to the patient's intolerance) with findings of non-specific colitis (with biopsies) and an esophagogastroduodenoscopy without pathological findings (without biopsies). Colonoscopy biopsies were not suggestive of a specific disease. The patient was treated empirically with antibiotics (ciprofloxacin, 500 mg b.i.d., and metronidazole, 500 mg t.i.d.) and a low fiber diet for a few days with partial remission of symptoms.

At presentation, the medication review revealed a prescription for olmesartan as antihypertensive therapy; thus, discontinuation was ordered immediately. The patient had signs of dehydration on physical examination, the abdomen was distended with slight tenderness in the left lower quadrant with no rebound or guarding, and there were normal bowel sounds.

Laboratory tests revealed normocytic, normochromic anemia consisting of a hematocrit of 30.20%, hemoglobin of 10.20 g/dL, a mean corpuscular volume (MCV) of 87.30 fL, a mean corpuscular hemoglobin (MCH) of 27.70 pg, a mean corpuscular hemoglobin concentration (MCHC) of 31.80 g/dL, a low serum protein of 4.9 g/dL, an albumin of 2.2 g/dL, low serum calcium (Ca) of 7.6 mg/dL, and magnesium (Mg) of 1.2 mg/dL). The leukocyte count, coagulation, erythrocyte sedimentation rate, C-reactive protein, liver function tests, and serum thyroid-stimulating hormone were within the normal range. The rest of the laboratory tests were as follows: negative stool investigations (culture, parasitology, and Clostridium difficile toxins), negative human immunodeficiency virus (HIV) serology, normal serum protein electrophoresis, a normal 24-hour urine protein, antinuclear antibodies titer 1:320, negative anti-smooth muscle antibodies, negative antimitochondrial antibodies, negative anti-parietal cell antibodies, normal immunoglobulin G (IgG), immunoglobulin A (IgA), and immunoglobulin M (IgM) levels, and negative celiac serology (anti-endomysial antibodies and anti-tissue transglutaminase IgG and IgA).

**Figure 1:**
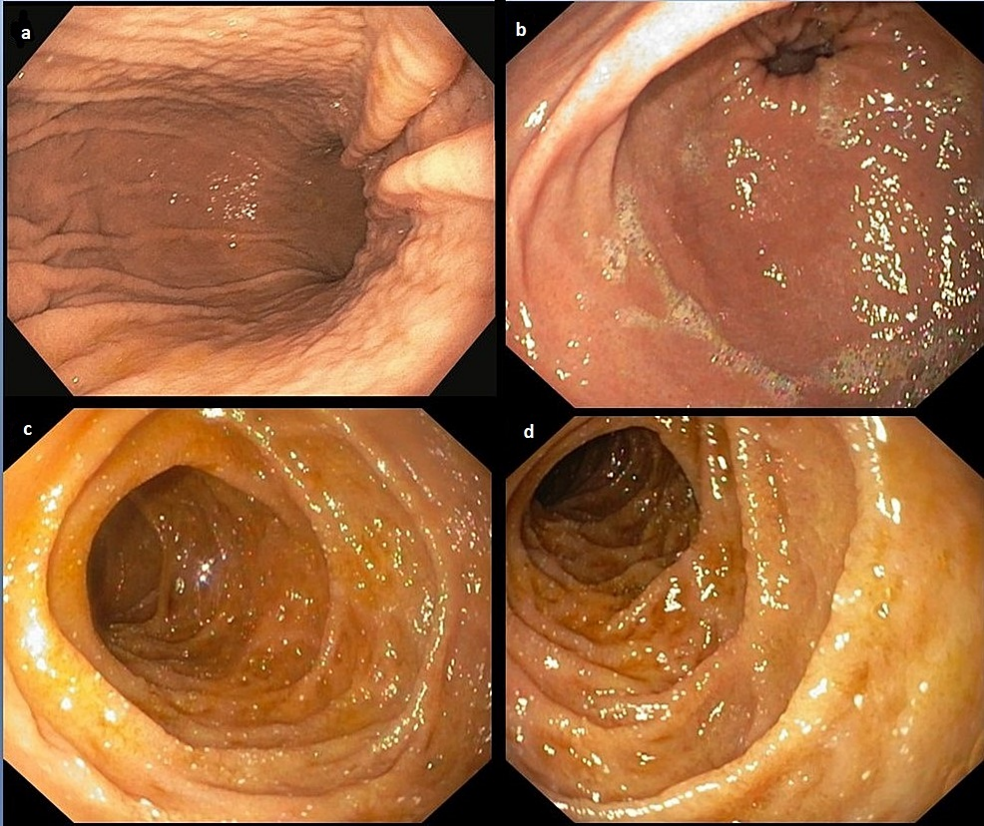
Upper gastrointestinal endoscopy This study shows swelling and redness of the mucosa of the stomach (a, b) with flattening of the villi and scalloping of the duodenum (c, d).

A repeat upper and lower endoscopy was performed with biopsies. The upper endoscopy (Figure 1) showed swelling and redness of the mucosa of the stomach (Figure 1a-b) with flattening of the villi and scalloping of the duodenum (Figure 1c-d).

**Figure 2:**
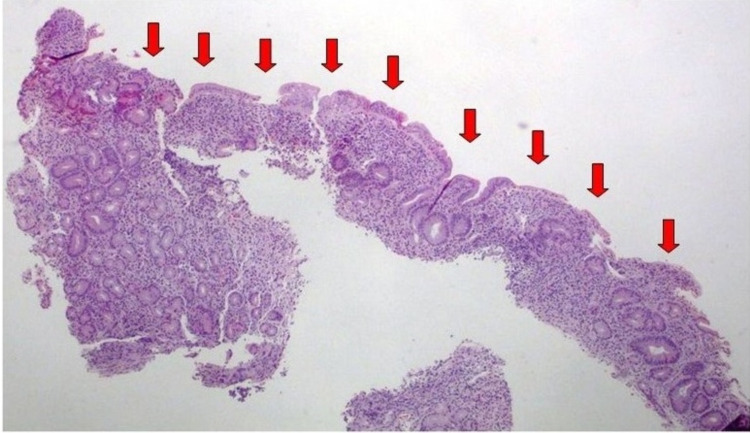
Histopathological evaluation of duodenal mucosal biopsies showing severe villous atrophy (red arrows) Hematoxylin and eosin staining 4x

**Figure 3:**
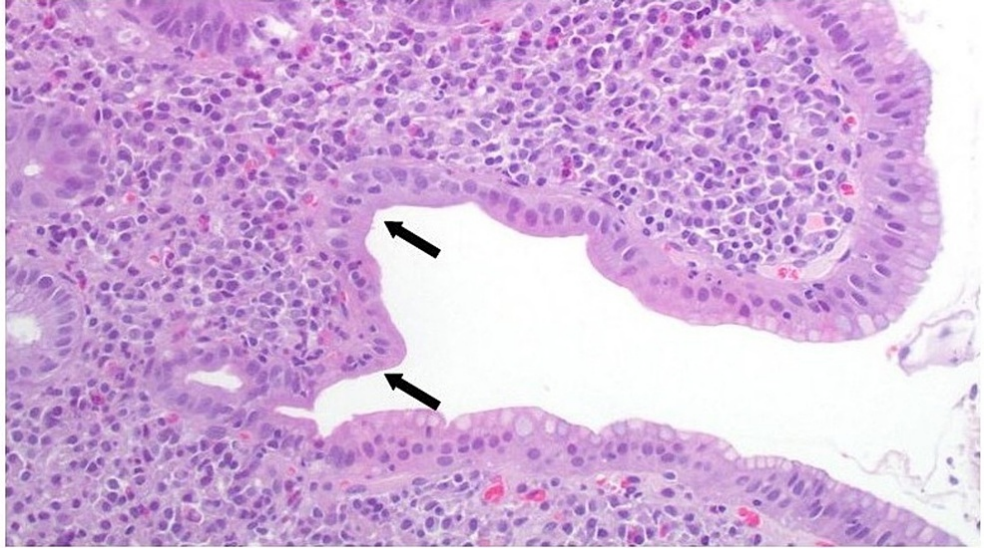
Histopathological evaluation of duodenal mucosal biopsies showing chronic inflammatory infiltration of the lamina propria and mucosal granulocytic infiltration (black arrows) Hematoxylin and eosin staining 100x

**Figure 4:**
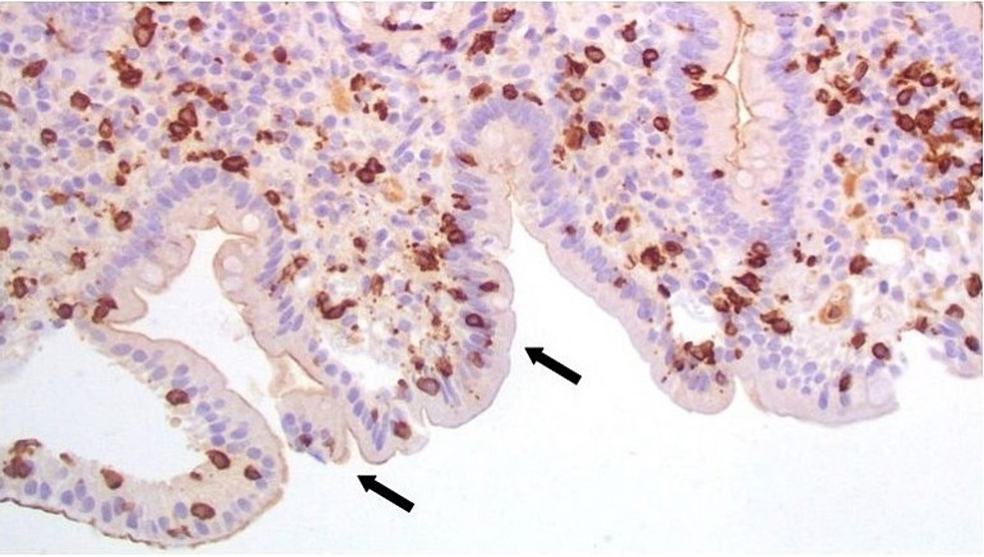
Histopathological evaluation of the duodenal mucosal biopsies showing mild intraepithelial lymphocytosis (black arrows) Immunohistochemical staining cluster of differentiation (CD)3/CD8 T cells; 100x

Biopsies from the duodenum revealed severe villous atrophy (Figure 2), chronic inflammatory infiltration of the lamina propria (Figure 3), and mild intraepithelial lymphocytosis (Figure 4). Gastric mucosal biopsies demonstrated chronic active lymphocytic gastritis without detection of Helicobacter pylori organisms.

**Figure 5:**
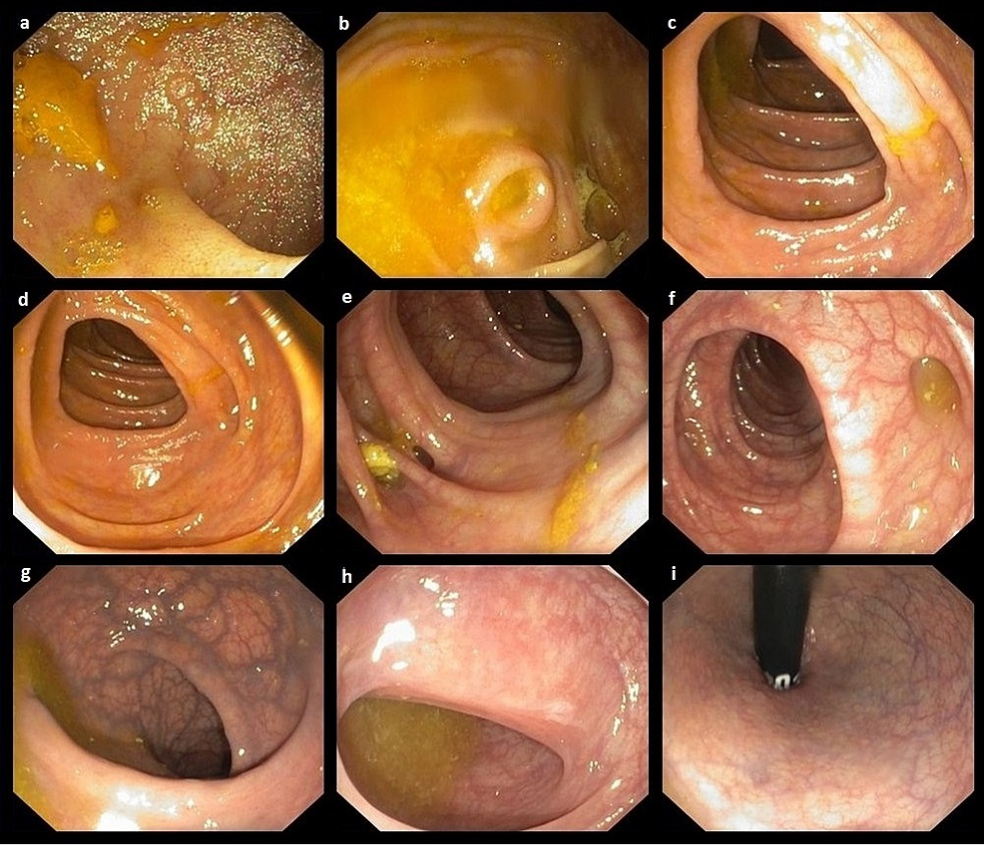
Lower gastrointestinal colonoscopy A lower gastrointestinal colonoscopy shows the ileum (a), cecum (b), and ascending and transverse colon (c, d) with no obvious pathology. There is diverticulosis of the descending and sigmoid colon (e, f), rectum with phlebectasia (g, h), and the anal canal (i) with no pathology.

Colonoscopy (Figure 5) showed diverticulosis of the descending and sigmoid colon (Figure 5e-f) without inflammatory or neoplastic changes. The colonic mucosal biopsies revealed microscopic colitis with focal intraepithelial lymphocytosis and chronic inflammatory lymphoplasmacytic infiltration of the lamina propria.

During hospitalization, while on a low-residue diet and after the discontinuation of olmesartan, the patient showed rapid symptomatic improvement. The patient was advised to avoid antihypertensive medication containing olmesartan, and a calcium channel blocker was used as an alternative antihypertensive drug with equal results. After a few days of hospitalization, the patient’s symptoms significantly resolved, blood electrolytes and protein levels were restored, and the patient was discharged home. Two months later, the patient reported no recurrences and had regained a lot of the lost weight, while a repeat upper gastrointestinal endoscopy confirmed restoration of villi atrophy.

## Discussion

Olmesartan is a widely used drug for the treatment of high blood pressure [3]. OIE was first described by Rubio-Tapia et al. in 2012 [1-2, 6-8]. Since then, several case reports and small case series have been documented [3, 6]. Due to its rarity, the lack of awareness of the disease commonly results in delayed diagnosis [9]. Symptoms of enterop­athy can be disabling for patients, and physicians may resort to extensive testing to diagnose the etiology of diarrhea and weight loss [4]. As a result, patients are usually subjected to various investigations and empirical treatment without response [9].

OIE can mimic other diseases, and physicians must be aware of this condition in order to avoid unnecessary investigations and delays in the diagnosis [1]. Drug-induced, sprue-like enteropathy must be considered as a potential diagnosis of patients presenting with diarrhea, severe weight loss, and villous atrophy of the duodenal mucosa of unknown origin [3]. Differential diagnosis should also include drug-related enteropathies, celiac disease, small-bowel bacterial overgrowth, parasitic infestations, hypogammaglobulinemia sprue, giardiasis, autoimmune enteropathy, tropical sprue, collagenous sprue, intestinal lymphoma, HIV-related enteropathy, Whipple's disease, and unclassified sprue [1, 3-4, 6, 8].

The pathogenesis of the syndrome remains unclear [3]. One of the proposed mechanisms involves a cell-mediated immune response that damages the small intestinal brush border [2-3]. In addition, it is thought that the ARB class of drugs has an inhibitory action of transforming growth factor-beta (TGF-β), which is important for the gut homeostasis and apoptosis of enterocytes due to disproportionate activation of type 2 angiotensin II (AT2) receptors after blockage of type 1 angiotensin II receptors (AT1) [2, 4-6, 8].

In individuals taking olmesartan, the mere dis­continuation of the medication leads to prompt resolution of the symptoms [4]. Olmesartan discontinuation results in rapid symptomatic improvement within days to few weeks, while histological recovery is observed within a few months [9]. Confirmation of the diagnosis requires clinical resolution of the symptoms after olmesartan discontinuation and suggestive gastrointestinal histological findings [1]. Villous atrophy (total or partial) is almost always present in duodenal biopsies and gastric and colon biopsies may demonstrate lymphocytic gastritis and microscopic colitis, respectively [9].

We first suspected the possible connection between enteropathy and olmesartan when the patient was referred to the emergency department reporting severe chronic diarrhea while on antihypertensive treatment including olmesartan. Our patient had clinical features suggestive of celiac disease; how­ever, the initial assessment revealed negative serology. An upper gastrointestinal endoscopy was done subsequently showing marked villous atrophy. Olmesartan was discontinued from the beginning and the patient’s symptoms resolved gradually. A re-challenge with olmesartan was not done as the patient’s symptoms were very distressing. The fact that a thorough investigation for all the known conditions for chronic diarrhea was negative and that her symptoms improved after discontinuing the olmesartan led us to conclude that this was a case of OIE.

We report a case to support a novel association between severe sprue-like enteropathy and olmesartan, as well as to alert physicians to the importance of timely recognition of this cause of sprue-like enteropathy.

## Conclusions

Although olmesartan-induced enteropathy is a rare condition, it is associated with a severe diarrheal syndrome. Thus, cautious medication review is imperative and an extensive investigation for chronic diarrhea in patients taking olmesartan should be avoided. Initially, they should be given an olmesartan drug-free period to verify if symptoms resolve prior to pursuing further evaluation. Increased awareness of this condition may spare affected patients of unnecessary diagnostic tests and treatments as symptoms resolve within days after olmesartan discontinuation.
